# Prognostic relevance of remission and measurable residual disease status in AML patients prior to reduced intensity or non-myeloablative allogeneic stem cell transplantation

**DOI:** 10.1038/s41408-021-00471-x

**Published:** 2021-04-29

**Authors:** Madlen Jentzsch, Juliane Grimm, Marius Bill, Dominic Brauer, Donata Backhaus, Julia Schulz, Karoline Goldmann, Dietger Niederwieser, Uwe Platzbecker, Sebastian Schwind

**Affiliations:** Medical Clinic and Policlinic 1, Hematology, Cellular Therapy and Hemostaseology, Leipzig University Hospital, Leipzig, Germany

**Keywords:** Acute myeloid leukaemia, Acute myeloid leukaemia

Dear Editor,

Acute myeloid leukemia (AML) presents highly heterogeneous, calling for individualized treatment approaches. Allogeneic hematopoietic stem cell transplantation (HSCT) offers the consolidation treatment with the highest chance of sustained remission for most AML patients^1^. In patients refractory to induction therapy or suffering early relapse, allogeneic HSCT may be performed as a salvage therapy despite the detection of active disease^2^.

At AML diagnosis the European LeukemiaNet (ELN) 2017 risk stratification identifies three prognostically relevant groups, also in patients receiving allogeneic HSCT for consolidation^1,3^. Measurable residual disease (MRD) evaluation at various time points during the disease course, including prior to HSCT, has been shown to provide valuable additional risk stratification in AML patients independently of the applied MRD marker and method^4–9^. Importantly, in an AML cohort receiving allogeneic HSCT, one study showed that outcomes of MRD-positive (MRD^pos^) patients were similarly dismal as in patients transplanted with morphologic active disease^10^. However, the median age in this analysis was 50 years, and all patients received myeloablative (MAC) conditioning, leaving open questions regarding individuals not eligible for intensive conditioning therapies. Here, reduced intensity (RIC) or non-myeloablative (NMA) conditioning is being applied where disease control increasingly relies on immunological graft-versus-leukemia (GvL) effects^11^.

To compare the prognostic significance of the presence of a morphologic remission and the MRD status prior to performing RIC- or NMA-HSCT, as well as the impact of GvL effects, we retrospectively analyzed 392 AML patients who received an allogeneic HSCT. All patients received NMA- (74%) or RIC-HSCT (26%) at a median age of 63.1 (range 21.4–76.8) years with either active disease (33%) or in morphologic complete remission (CR) or CR with incomplete peripheral recovery (CRi, 67%). Details on the applied conditioning regimens are given in the Supplementary Information. Patients’ characteristics are shown in Supplementary Table S1. Median follow-up after HSCT was 2.8 years. Written informed consent was obtained from all patients in accordance with the Declaration of Helsinki. MRD status at HSCT was evaluated for all patients transplanted in CR/CRi using quantitative polymerase chain reaction for at least one of the targets *NPM1* mutation, *BAALC/ABL1*, *MN1/ABL1*, or *WT1/ABL1* expression adapting the previously published cut-offs^5–7^. Patients with at least one positive test result were regarded as MRD^pos^.

After NMA- or RIC-HSCT patients transplanted in MRD^neg^ remission had the best outcomes which also remained significant in multivariate analyses (Supplementary Table S2). With respect to the cumulative incidence of relapse (CIR) rates, our data resembled those following MAC-HSCT^10^, with equally high CIR rates around 50–60% in patients transplanted with MRD^pos^ or active disease (Supplementary Fig. S1A). These results—despite different MRD assessment methods (molecular vs flow-based)—resemble those of Araki et al. However, while non-relapse mortality (NRM) was comparable, overall survival (OS) and event-free survival (EFS) were significantly longer in MRD^pos^ patients compared to those transplanted with active disease (Supplementary Fig. S1B, S1C and Fig. 1A). This indicates that some MRD^pos^ patients may be salvaged following relapse after HSCT and may achieve long-term survival even when transplanted in a suboptimal remission state.

**Fig. 1:**
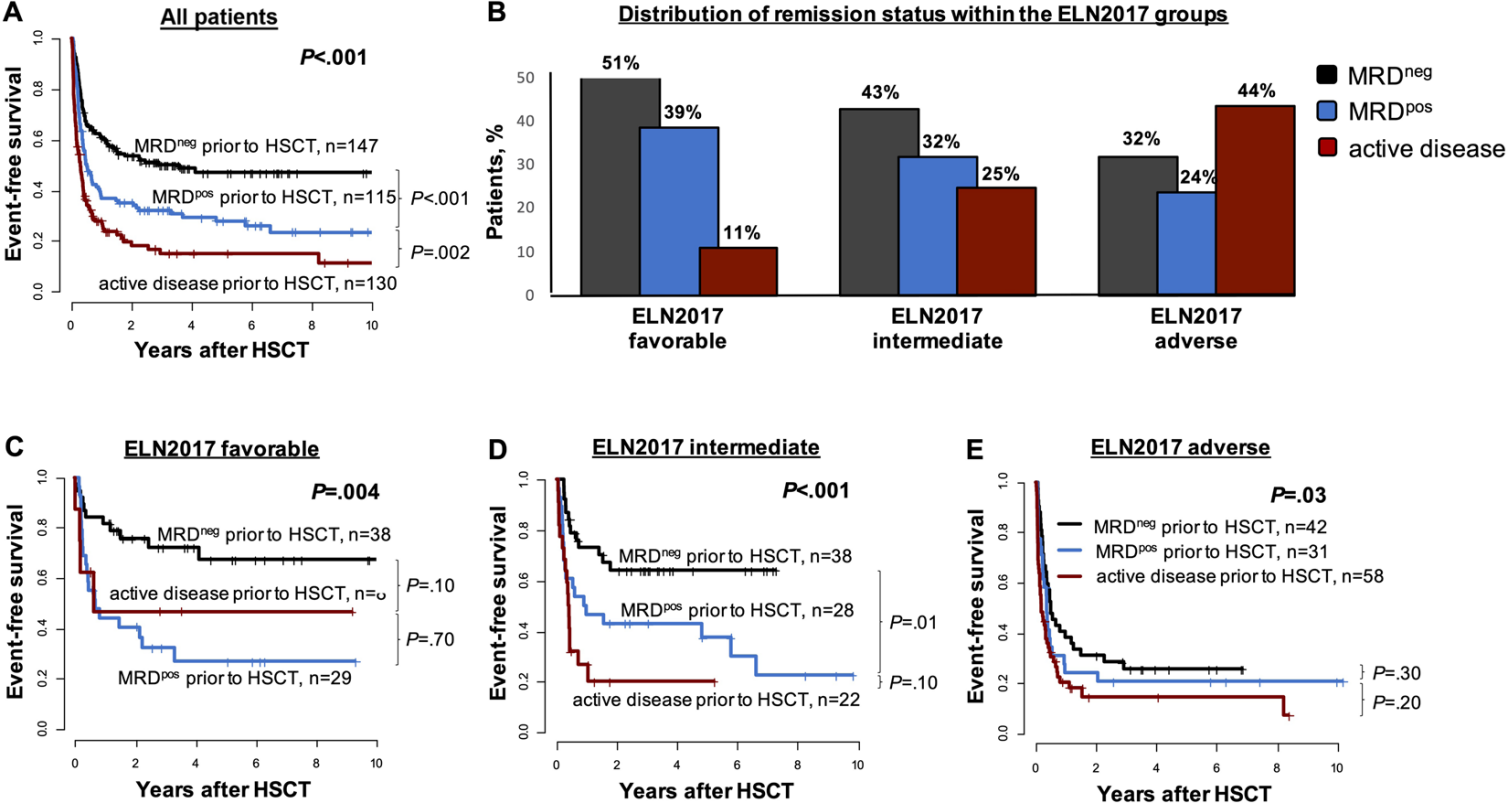
Outcome and ELN2017 risk distribution according to remission status prior to allogeneic RIC- or NMA-HSCT (MRD^neg^ vs MRD^pos^*vs* active disease, *n* = 392). **A** Event-free survival of the whole patient cohort, **B** distribution of the remission status prior to allogeneic RIC- or NMA-HSCT according to the ELN2017 risk stratification system, **C** event-free survival in ELN2017 favorable-risk patients (*n* = 75), **D** event-free survival in ELN2017 intermediate-risk patients (*n* = 88), and **E** event-free survival in ELN2017 adverse risk patients (*n* = 131).

In our study, the applied conditioning regimens differed significantly between patients with MRD^neg^, MRD^pos^, and active disease prior to HSCT, mostly because FLAMSA RIC-HSCT was purposely administered to individuals with the highest relapse risk, usually identified by an active disease prior to allogeneic HSCT. Consequently, we performed a subanalysis that included only individuals receiving the conditioning with the lowest intensity and without thymoglobulin (NMA, 74% of our cohort) where we yielded similar results as in the whole patient cohort (Supplementary Fig. S2). These data and the observed comparable outcomes between RIC and NMA conditioned patients transplanted with active disease (Supplementary Fig. S3) indicate that our results are rather independent of the applied conditioning regimen. Thus, the deepest possible remission is necessary prior to HSCT to improve outcomes and should be evaluated as part of individualized prognostication and treatment decisions.

We also separately analyzed the three ELN2017 risk groups. The distribution of the patients within the ELN2017 groups reflected the phenotype aggressiveness and the associated difficulties achieving a deep remission prior to HSCT. While there was a stepwise decrease of MRD^neg^ and MRD^pos^ patients, the number of patients transplanted with the active disease increased from ELN2017 favorable to intermediate to adverse risk (Fig. 1B). We observed the strongest outcome separation between MRD^neg^, MRD^pos^, and active disease at HSCT in patients with favorable and intermediate ELN2017 risk AML (Fig. 1C–E and Supplementary Fig. S4): MRD^neg^ patients had significantly longer EFS in favorable (*P* = 0.004) and intermediate (*P* < 0.001). ELN2017 risk compared to those with MRD^pos^ or active disease. In ELN2017 adverse risk patients, MRD^neg^ patients also had improved EFS compared to those with MRD^pos^ or active disease (*P* = 0.03), but EFS in general was short and the impact of the remission status at HSCT limited.

Intriguingly, a variety of parameters at diagnosis known to associate with worse outcomes in AML patients were already significantly different between patients transplanted with active disease compared to those in MRD^neg^ or MRD^pos^ remission. A higher incidence of secondary AML (*P* = 0.04), a higher genetic risk, including an abnormal (*P* < 0.001), monosomal (*P* < 0.001), or complex karyotype (*P* = 0.006), adverse ELN2017 genetic risk (*P* < 0.001), the presence of *TP53* mutations (*P* = 0.05), as well as the absence of *NPM1* mutations (*P* < 0.001) were more frequently found in patients transplanted with active disease (Supplementary Table S1). In contrast, MRD^neg^ and MRD^pos^ patients only differed regarding their white blood count and *SRSF2* mutation status, which again underlines the importance of a dynamic risk stratification during remission. Thus, MRD assessment is especially important in lower or intermediate-risk AML patients, which of course is also true regarding potential consolidation decisions towards allogeneic HSCT.

Since RIC and NMA conditioning regimens rely on GvL effects for disease control we performed a landmark analysis of patients surviving longer than 100 days after HSCT to evaluate the prognostic impact of the presence of chronic graft versus host disease (GvHD) as a known surrogate marker for GvL effects. In the entire set the presence of chronic GvHD favorably impacted outcomes following RIC- or NMA-HSCT in univariate and multivariate analyses (Supplementary Table S3). We observed longer OS and EFS for MRD^pos^ patients compared to patients transplanted with active disease, as well as a favorable effect of chronic GvHD in patients with MRD^neg^, by trend in patients with MRD^pos^, but no effect in patients transplanted with active disease (Fig. 2 and Supplementary Fig. S5). The GvL impact was reduced in MRD^pos^ and more or less lost in the group of patients transplanted with active disease. Thus, the strengths of the GvL effect seem to depend on the disease burden at HSCT and may unfold its full potential only in patients with a low disease burden (i.e., MRD^neg^) to help control AML following HSCT. This observation may also contribute to the outcome differences observed for the different remission status following HSCT.

**Fig. 2:**
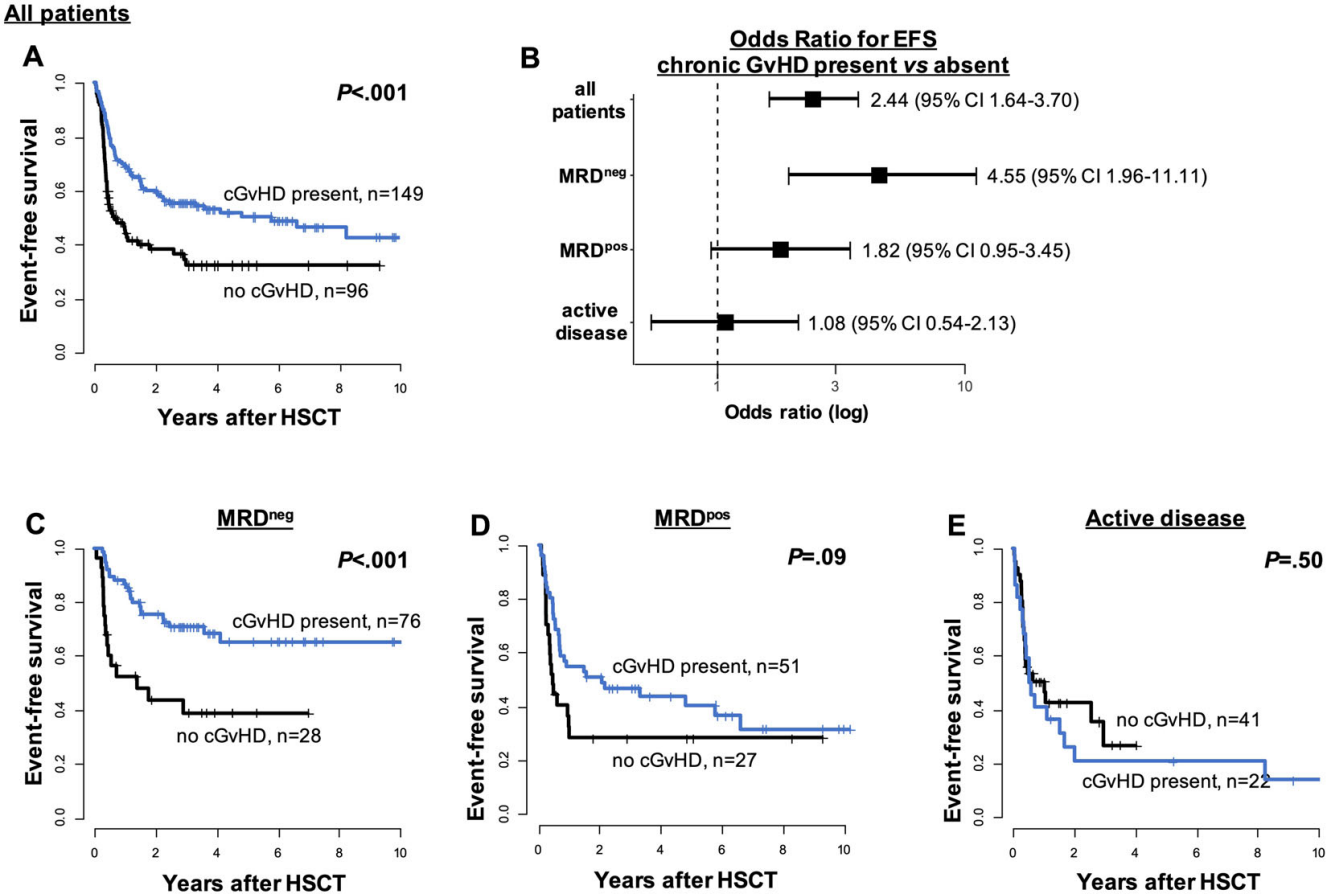
Event-free survival according to the presence of a chronic GvHD in patients surviving longer than 100 days after allogeneic RIC- or NMA-HSCT (landmark analysis). **A** All patients (*n* = 245), **B** forest-plot for the odds-ratio for EFS when chronic GVHD is present for all patients and for the subgroups according to the remission status, **C** EFS according to the presence of chronic GvHD in MRD^neg^ patients (*n* = 104), **D** MRD^pos^ patients (*n* = 78), and **E** patients with active disease (*n* = 63) at HSCT.

Today some clinical trials have addressed the remission depth before HSCT in AML. Application of additional therapies in AML patients not in remission at HSCT may introduce deeper responses and longer survival, which is currently evaluated in a prospective clinical trial (ETAL3-ASAP, NCT02461537). Also, the choice of the conditioning regimen and immunosuppression might be helpful in improving outcomes. MRD^pos^ patients may benefit from more intensive conditioning regimen^12^, application of donor lymphocytes (Supplementary Fig. S6), or the absence of T-cell depletion (Supplementary Fig. S7)^13^ which of course has to be carefully weighed against a potentially higher NRM. Also, donor selection may contribute to improved outcomes of MRD^pos^ patients, as in previous studies the use of haploidentical donors resulted in better disease control, longer survival, and similar NRM than sibling HSCT in patients transplanted with active disease^14^ or MRD^pos^ remission^15^.

With respect to the ELN2017 adverse group the observed outcomes, irrespective of the morphologic remission or MRD status are sobering. The very abysmal outcomes of ELN2017 adverse risk patients following HSCT call for novel treatment approaches and these patients should be entered into clinical trials whenever possible. Some hope also relies on novel drug combinations that may induce a deeper remission before HSCT and/or approaches regarding maintenance after HSCT.

In their paper, Araki et al. also raised the question of routine use of refined remission criteria to include more sensitive methods, such as the “complete remission without MRD”^9^. With the here presented data we second this suggestion. Certainly, clinical trials prospectively testing risk-adapted treatment algorithms are needed to change and individualize routine clinical approaches.

In conclusion, our study is the first to indicate comparable high relapse rates in MRD^pos^ patients and patients receiving RIC- or NMA-HSCT with active disease. MRD^neg^ patients at HSCT had the best outcomes, an effect that is most pronounced in the ELN2017 favorable and intermediated risk groups. Following RIC or NMA conditioning the GvL effect seems to have the highest impact in patients with a low disease burden at HSCT. The morphologic remission and MRD status at HSCT are prognostically very important in AML patients receiving RIC- or NMA-HSCT and should routinely be assessed to improve individualized prognostication.

## Supplementary information

Supplementary Information

AJ-Checklist
