# Supplementary material for: Prognostic relevance of remission and measurable residual disease status in AML patients prior to reduced intensity or non-myeloablative allogeneic stem cell transplantation
Source: Blood Cancer J. 2021 Apr 29;11(4):80. doi: 10.1038/s41408-021-00471-x (PMC8084997; doi:10.1038/s41408-021-00471-x)
Supplement: Supplementary file 1 — Supplementary Information [file 41408_2021_471_MOESM1_ESM.docx]

***Supplementary Information***

**Prognostic relevance of remission and measurable residual disease status in AML patients prior to reduced intensity or non-myeloablative allogeneic stem cell transplantation**

Jentzsch M, *et al.*

**Patients’ characteristics**

All patients received a non-myeloablative (NMA) or reduced intensity conditioning (RIC) allogeneic hematopoietic stem cell transplantation (HSCT) between July 1998 and December 2019 at the University Hospital Leipzig. Clinical, genetic, and flow cytometry-related information at diagnosis and HSCT-related characteristics are shown in Supplementary Table S1. The data was analyzed in agreement with a bio banking registry of treated acute myeloid leukemia (AML) patients in our institution, which was approved by the local IRB.

**Allogeneic HSCT**

The majority of patients (n=290; 74%) received NMA-HSCT with 3x30 mg/m^2^ Fludarabine and 2 Gy (three patients received 3x30 mg/m^2^ Fludarabine and 3 Gy and five patients received 2 Gy alone) total body irradiation (TBI). 102 patients (26%) received RIC, either with combinations of Fludarabine, Cytarabine, and Amsacrine (FLAMSA, n=80) as previously described,^1^ or with 5x30 mg/m^2^ Fludarabine combined with either 140 mg/m^2^ Melphalan (n=7),^2^ or Busulfan (8mg/kg orally or 6.4 mg/kg intravenously, n=15).^3^

**Immunosuppression and graft-versus-host disease**

For prevention of graft-versus-host disease (GvHD), all patients received an intravenous starting dose of 5 mg/kg body weight cyclosporine A in two daily doses from day -1 which was adjusted to a whole-blood target level of 120-150 ng/ml for patients receiving FLAMSA conditioning or 200 ng/ml for all others.

Patients undergoing NMA-HSCT additionally received mycophenolate mofetil 3 g per day in three daily doses in case of unrelated HSCT or 2 g per day in two daily doses in case of related HSCT. None of the patients undergoing NMA-HSCT received *in vivo* T-cell depletion.

Patients receiving FLAMSA conditioning additionally received 2 g mycophenolate mofetil per day, which was stopped at day 28. Patients transplanted after RIC additionally received methotrexate 15 mg intravenously on days +1, +3, +6, and +11 after HSCT, and RIC patients transplanted from an unrelated donor additionally received *in vivo* T-cell depletion with thymoglobulin 2 mg/kg per day for three days. Cyclosporine A was reduced starting on day +42 and stopped on day 120 following FLAMSA conditioning and for all others reduced starting on day +84 or day +180 following related or unrelated HSCT, respectively. After NMA conditioning, mycophenolate mofetil was stopped at day +28 following related HSCT and tapered from days +40 to +96 following unrelated HSCT.^6^ Patients were evaluated for incidence of acute GvHD and chronic GvHD using established criteria of the Glucksberg grading system.^4^ Immunosuppression was prolonged or extended with systemic steroids in cases of GvHD (grade > 2 according to Glucksberg grading system).^4^ Requirement for acute GvHD was engraftment while requirement for chronic GvHD was engraftment and survival for at least 100 days after HSCT.

In patients receiving FLAMSA conditioning who did not develop signs of a GvHD, up to three prophylactic donor lymphocyte infusions (DLI) were administered per protocol after day 120 in increasing doses (5 x 10^5^/kg, 5 x 10^6^/kg, and 1 x 10^7^/kg recipient weight for patients receiving unrelated HSCT and 1 x 10^6^/kg, 5 x 10^6^/kg, and 1 x 10^7^/kg recipient weight for patients receiving related HSCT).

**Definition of complete remission and active disease**

Complete remission (CR) was defined as the presence of <5% of blasts in bone marrow, neutrophils >1.0 x 10^9^/L, platelets >100 x 10^9^/L, absence of blasts with Auer rods, independence of blood transfusion and no extramedullary disease.^5^ CR with incomplete peripheral recovery (CRi) was defined as CR with platelets <100 x 10^9^/L or neutrophils <1.0 x 10^9^/L. In patients receiving allogeneic HSCT, the presence of CR or CRi was confirmed within 28 days prior to HSCT by bone marrow and peripheral blood analysis. Active disease at HSCT was defined by a persisting blast count >5% in bone marrow, persisting blasts in peripheral blood or the detection of extramedullary disease.

**Flow Cytometry, Cytogenetics, and Molecular Markers**

Bone marrow mononuclear cells at diagnosis were assessed for surface presence of an institutional standard panel as previously described.^6,7^ Cytogenetic analyses at diagnosis were performed using standard techniques of banding and *in situ* hybridization. The mutation status of the genes CCAAT/enhancer-binding protein alpha (*CEBPA*) and nucleophosmin 1 (*NPM1)* as well as the presence or absence of an internal tandem duplications in the *FLT3* gene (*FLT3*-ITD) were evaluated as previously described.^6^ For patients with pretreatment material available, the mutation status of 54 genes included in the TruSight Myeloid Sequencing Panel (Illumina, San Diego, CA, USA) was evaluated using next generation sequencing (NGS) at diagnosis as previously described.^8^ *ASXL1* mutations at codon 646 were validated applying a proof-reading polymerase based Sanger sequencing approach.^8^ Patients were grouped according to the ELN2017 recommendations.^9^

**MRD Assessment prior to allogeneic HSCT**

Measurable residual disease (MRD) status at HSCT was evaluated for all patients transplanted in CR/CRi using digital droplet polymerase chain reaction (PCR) for targets *NPM1* mutation, *BAALC/ABL1*, or *MN1/ABL1* copy numbers as well as quantitative real time PCR for the target *WT1/ABL1* expression adapting the previously published cut-offs.^10–13^ Patients with at least one positive test result were regarded as MRD^pos^.

The MRD status at HSCT relied on one marker in 32 patients, on two markers in 54 patients, on three markers in 149 patients and on all four markers in 27 patients. Of note, since this is a retrospective study treating physicians usually did not have access to MRD results with the exception of *NPM1* MRD for patients transplanted after May 2015 (n=22) after introduction of the assay into the clinical routine.

**Statistical Analyses**

Overall survival (OS) and event-free survival (EFS) were calculated from HSCT until death from any cause and relapse or death from any cause, respectively. Survival estimates were calculated using the Kaplan-Meier method and groups were compared using the log-rank test. The competing risks cumulative incidence of relapse (CIR) and non-relapse mortality (NRM) were calculated from HSCT to relapse/progression or death, respectively, using the Fine and Gray method.^14^ Associations with baseline clinical, demographic and molecular features were compared using the Kruskal-Wallis-Test and Fisher’s exact tests for continuous and categorical variables, respectively. All statistical analyses were performed using the R statistical software platform (version 3.4.3).^15^

Multivariable proportional hazard models were constructed for CIR, NRM, EFS, and OS to evaluate the impact of the MRD-corrected remission status prior to HSCT in patients receiving RIC or NMA conditioning by forward adjusting for other variables. The following variables were considered for multivariable analyses: sex, disease origin (*de novo* vs secondary), European LeukemiaNet (ELN) 2017 risk, mutation status of the tyrosine kinase domain of the *FLT3* gene (*FLT3*-TKD), age at HSCT, disease status at HSCT (MRD^neg^ *vs* MRD^pos^ *vs* active disease), number of remission (first *vs* second *vs* none), conditioning regimen (RIC *vs* NMA), cytomegalovirus (CMV) status of recipient and donor (high-risk [+/-] *vs* all others), donor type (matched related *vs* matched unrelated *vs* mismatched unrelated), and sex of the donor (female into male *vs* all others). Of these, variables significant at α=.10 in univariable analyses were considered for multivariable analyses. For all endpoints, hazard/odds ratios with their corresponding 95% confidence intervals are indicated for every significant prognostic factor of the final model.

**Multivariate landmark analyses in patients surviving longer than 100 days after HSCT**

Additional to the whole patient cohort we performed landmark multivariate analyses of patients surviving longer than 100 days after HSCT and added the presence *vs* absence of chronic GvHD as a covariate into the models. Similar to the analyses for the whole patient cohort, the MRD corrected remission status prior to HSCT remained a significant factor for CIR, OS, and EFS (Supplementary Table S3).

**Prognostic impact of application of DLI**

Preemptive DLIs according to protocol were planned in patients receiving FLAMSA-based conditioning (n=80). Of those, 49 were alive and relapse free at day 120 and eligible to receive up to three preemptive DLIs per protocol. Of those patients, only three were MRD^neg^ prior to HSCT (one received a DLI), only one was MRD^pos^ prior to HSCT (and did not receive a DLI). All others were transplanted with active disease, preventing separate analyses according to the remission status at HSCT. Regarding all FLAMSA transplanted patients alive at days 120 after HSCT, the administration of DLIs associated with a lower CIR (*P*=.05), shorter EFS (*P*=.008), and by trend shorter OS (*P*=.06, Supplementary Figure S6).

**Prognostic impact of *in vivo* T-cell depletion**

Previously, Dillon *et al.* suggested that MRD^pos^ patients at HSCT might benefit from conditioning regimens without T-cell depletion.^16^ In our patients set, only patients receiving RIC-HSCT from an unrelated donor received *in vivo* T-cell depletion with thymoglobulin (n=93). When we restricted the analysis to this patient population, outcomes of MRD^pos^ patients were dismal and comparable to patients transplanted with active AML (Supplementary Figure S7). These results are consistent with the data of Dillon *et al.*^16^ However, our analysis is limited by low patient numbers and should be interpreted with caution.

**Supplementary References**

1 Pfrepper C, Klink A, Behre G, Schenk T, Franke G-N, Jentzsch M *et al.* Risk factors for outcome in refractory acute myeloid leukemia patients treated with a combination of fludarabine, cytarabine, and amsacrine followed by a reduced-intensity conditioning and allogeneic stem cell transplantation. *J Cancer Res Clin Oncol* 2016; **142**: 317–324.

2 Bryant A, Nivison-Smith I, Pillai ES, Kennedy G, Kalff A, Ritchie D *et al.* Fludarabine Melphalan reduced-intensity conditioning allotransplanation provides similar disease control in lymphoid and myeloid malignancies: Analysis of 344 patients. *Bone Marrow Transplant* 2014; **49**: 17–23.

3 Kröger N, Iacobelli S, Franke GN, Platzbecker U, Uddin R, Hübel K *et al.* Dose-reduced versus standard conditioning followed by allogeneic stem-cell transplantation for patients with myelodysplastic syndrome: A prospective randomized phase III study of the EBMT (RICMAC Trial). *J Clin Oncol* 2017; **35**: 2157–2164.

4 Glucksberg H, Storb R, Fefer A, Buckner CD, Neiman PE, Clift RA *et al.* Clinical manifestations of graft-versus-host disease in human recipients of marrow from HL-A-matched sibling donors. 1974; : 295–304.

5 Döhner H, Estey EH, Amadori S, Appelbaum FR, Büchner T, Burnett AK *et al.* Diagnosis and management of acute myeloid leukemia in adults: Recommendations from an international expert panel, on behalf of the European LeukemiaNet. *Blood* 2010; **115**: 453–474.

6 Jentzsch M, Bill M, Grimm J, Schulz J, Schuhmann L, Brauer D *et al.* High expression of the stem cell marker GPR56 at diagnosis identifies acute myeloid leukemia patients at higher relapse risk after allogeneic stem cell transplantation with the CD34+/CD38- population. *Haematologica* 2020; **105**: e507.

7 Jentzsch M, Bill M, Nicolet D, Leiblein S, Schubert K, Pless M *et al.* Prognostic impact of the CD34+/CD38− cell burden in patients with acute myeloid leukemia receiving allogeneic stem cell transplantation. *Am J Hematol* 2017; **92**: 388–396.

8 Grimm J, Bill M, Jentzsch M, Beinicke S, Häntschel J, Goldmann K *et al.* Clinical impact of clonal hematopoiesis in acute myeloid leukemia patients receiving allogeneic transplantation. *Bone Marrow Transplant* 2019; **54**. doi:10.1038/s41409-018-0413-0.

9 Döhner H, Estey E, Grimwade D, Amadori S, Appelbaum FR, Ebert BL *et al.* Diagnosis and management of AML in adults: 2017 ELN recommendations from an international expert panel. *Blood* 2017; **129**: 424–447.

10 Bill M, Grimm J, Jentzsch M, Kloss L, Goldmann K, Schulz J *et al.* Digital droplet PCR-based absolute quantification of pre-transplant NPM1 mutation burden predicts relapse in acute myeloid leukemia patients. *Ann Hematol* 2018; **97**: 1757–1765.

11 Jentzsch M, Bill M, Grimm J, Schulz J, Goldmann K, Beinicke S *et al.* High BAALC copy numbers in peripheral blood prior to allogeneic transplantation predict early relapse in acute myeloid leukemia patients. *Oncotarget* 2017; **8**: 87944–87954.

12 Jentzsch M, Bill M, Grimm J, Schulz J, Beinicke S, Häntschel J *et al.* Prognostic Impact of Blood MN1 Copy Numbers Before Allogeneic Stem Cell Transplantation in Patients With Acute Myeloid Leukemia. *HemaSphere* 2019; **3**: e167.

13 Lange T, Hubmann M, Burkhardt R, Franke GN, Cross M, Scholz M *et al.* Monitoring of WT1 expression in PB and CD34 donor chimerism of BM predicts early relapse in AML and MDS patients after hematopoietic cell transplantation with reduced-intensity conditioning. *Leukemia* 2011; **25**: 498–505.

14 Gray RJ. A Class of K-Sample Tests for Comparing the Cumulative Incidence of a Competing Risk. *Ann Stat* 1988; **16**: 1141–1154.

15 R Development Core Team. R: A language and environment for statistical computing. Vienna, Austria. 2017. doi:R Foundation for Statistical Computing, Vienna, Austria. ISBN 3-900051-07-0, URL http://www.R-project.org.

16 Dillon R, Hills R, Freeman S, Potter N, Jovanovic J, Ivey A *et al.* Molecular MRD status and outcome after transplantation in NPM1-mutated AML. *Blood* 2020; **135**: 680–688.

**Supplementary Tables**

**Supplementary Table S1.** Patients’ characteristics according to the remission status prior to HSCT (n=392).

|  | **All patients**  **n=392** | **CR/CRi MRD^neg^**  **n=147** | **CR/CRi MRD^pos^**  **n=115** | ***P***  **(MRD^neg^ vs MRD^pos^)** | **Active disease**  **n=130** | ***P***  **(CR/CRi vs active disease)** |
| --- | --- | --- | --- | --- | --- | --- |
| **Clinical parameters at diagnosis** | | | | | | |
| Age at diagnosis, years  median (range) | 62.4 (20.7-76.5) | 63.9 (32.6-74.8) | 62.1 (29.7-76.5) | .37 | 60.9 (20.7-75.3) | .001 |
| Sex, n (%)  male  female | 193 (49)  199 (51) | 74 (50)  73 (50) | 58 (50)  57 (50) | 1 | 61 (47)  69 (53) | .52 |
| Disease origin, n (%)  secondary/treatment related  *de novo* | 160 (41)  232 (59) | 53 (36)  94 (64) | 44 (38)  71 (62) | .80 | 63 (48)  67 (52) | .04 |
| Hemoglobin, g/dL  median (range) | 8.9 (3.2-15.3) | 9.1 (3.2-14.4) | 6.6 (4.3-15.3) | .34 | 8.9 (3.8-14.2) | .90 |
| Platelet count, x 10^9^/L  median (range) | 65 (2-950) | 80 (3-950) | 65 (3-276) | .13 | 49 (2-547) | .19 |
| WBC, x 10^9^/L  median (range) | 5.9 (0.1-385) | 4.5 (0.6-160) | 13.6 (0.1-385) | .03 | 5.3 (0.5-325) | .40 |
| Blood blasts, %  median (range) | 20 (0-97) | 11 (0-97) | 22 (0-97) | .10 | 20 (0-93) | .78 |
| BM blasts, %  median (range) | 50 (3-100) | 50 (3-94) | 50 (10-95) | .91 | 46.1 (9-100) | .29 |
| **Genetic parameters at diagnosis** | | | | | | |
| Normal karyotype, n (%)  absent  present | 207 (57)  159 (43) | 70 (49)  72 (51) | 50 (48)  54 (52) | .90 | 87 (73)  33 (28) | <.001 |
| ELN2017 group, n (%)  favorable  intermediate  adverse | 75 (26)  88 (30)  131 (45) | 38 (32)  38 (32)  42 (36) | 29 (33)  28 (32)  31 (35) | 1 | 8 (9)  22 (25)  58 (66) | <.001 |
| Complex karyotype, n (%)  absent  present | 293 (83)  60 (17) | 117 (87)  18 (13) | 90 (87)  13 (13) | 1 | 86 (75)  29 (25) | .006 |
| Monosomal karyotype, n (%)  absent  present | 201 (79)  55 (21) | 126 (92)  11 (8) | 90 (88)  12 (12) | .38 | 85 (73)  32 (27) | <.001 |
| *NPM1*, n (%)  wild-type  mutated | 223 (74)  78 (26) | 85 (69)  39 (31) | 61 (66)  32 (34) | .66 | 77 (92)  7 (8) | <.001 |
| *CEBPA*, n (%)  wild-type  mutated | 230 (92)  21 (8) | 96 (88)  13 (12) | 78 (93)  6 (7) | .33 | 56 (97)  2 (3) | .18 |
| *FLT3*-ITD, n (%)  wild-type  mutated | 240 (79)  64 (21) | 97 (78)  27 (22) | 72 (76)  23 (24) | .75 | 71 (84)  14 (16) | .27 |
| *RUNX1*, n (%)  wild-type  mutated | 74 (84)  14 (16) | 39 (87)  6 (13) | 28 (82)  6 (18) | .75 | 7 (78)  2 (22) | .63 |
| *TP53*, n (%)  wild-type  mutated | 81 (91)  8 (9) | 41 (91)  4 (9) | 33 (97)  1 (3) | .38 | 7 (70)  3 (30) | .04 |
| *ASXL1*, n (%)  wild-type  mutated | 77 (88)  11 (13) | 39 (87)  6 (13) | 30 (88)  4 (12) | 1 | 8 (89)  1 (11) | 1 |
| *SRSF2*, n (%)  wild-type  mutated | 138 (87)  20 (23) | 58 (82)  13 (18) | 59 (95)  3 (5) | .03 | 21 (84)  4 (16) | .53 |
| *TET2*, n (%)  wild-type  mutated | 65 (76)  21 (24) | 34 (76)  11 (24) | 26 (76)  8 (24) | 1 | 5 (71)  2 (29) | 1 |
| *IDH1*, n (%)  wild-type  mutated | 163 (90)  19 (10) | 78 (93)  6 (7) | 58 (87)  9 (13) | .27 | 27 (87)  4 (13) | .75 |
| *IDH2*, n (%)  wild-type  mutated | 159 (86)  26 (14) | 71 (83)  15 (17) | 60 (88)  8(12) | .37 | 28 (90)  3 (10) | .75 |
| *DNMT3A*, n (%)  wild-type  mutated | 84 (72)  32 (28) | 36 (69)  16 (31) | 38 (78)  11 (22) | .38 | 10 (67)  5 (33) | .55 |
| **Immunophenotype at diagnosis** | | | | | | |
| CD34+/CD38- cell burden  median (range) | 1 (0-89) | 0.5 (0-44.5) | 1 (0-89) | .09 | 2 (0-52) | <.001 |
| BM CD34 expression at diagnosis, %  median (range) | 18.3 (0-97) | 13 (0-90) | 17 (0.2-97) | .30 | 27 (1.5-91) | .002 |
| BM CD38 expression at diagnosis, %  median (range) | 68 (0.5-98) | 73 (20-98) | 71 (0.5-98) | .72 | 62 (4-96) | .02 |
| BM CD117 expression at diagnosis, %  median (range) | 33.7 (0-96) | 33 (0-93) | 33 (1.5-96) | .76 | 35 (1.4-89) | .47 |
| BM CD7 expression at diagnosis, %  median (range) | 17 (1-94) | 17 (1-90) | 18 (2-94) | .89 | 15 (2-90) | .84 |
| BM CD56 expression at diagnosis, %  median (range) | 9 (0.5-93) | 10 (0.5-93) | 9 (0.5-93) | .86 | 10 (1-80) | .25 |
| BM Glycophorin expression at diagnosis, %  median (range) | 11 (0-90) | 11 (0-61) | 10 (0.5-90) | .70 | 15 (0-73) | .01 |
| BM CD2 expression at diagnosis, %  median (range) | 15 (1-89) | 15 (3-81) | 14 (1-89) | .90 | 17 (2-84) | .15 |
| BM CD11b expression at diagnosis, %  median (range) | 14 (0.5-97) | 12 (0.5-91) | 17 (0.5-97) | .04 | 16 (0.5-86) | .76 |
| BM CD13 expression at diagnosis, %  median (range) | 54 (0.5-97) | 49 (0.5-96) | 59 (3-97) | .01 | 56 (5-92) | .83 |
| BM CD33 expression at diagnosis, %  median (range) | 62 (3-98) | 62 (4-97) | 70 (3-98) | .10 | 54 (4-95) | .10 |
| BM CD15 expression at diagnosis, %  median (range) | 25 (2-94) | 21 (2-94) | 26 (2-88) | .66 | 27 (4-79) | .52 |
| BM CD65 expression at diagnosis, %  median (range) | 15 (0.5-91) | 15 (0.5-91) | 13 (0.5-83) | .51 | 16 (1-72) | .95 |
| BM CD14 expression at diagnosis, %  median (range) | 3 (0.5-74) | 2 (0.5-70) | 3 (0.5-74) | .21 | 3 (0.5-56) | .21 |
| BM CD64 expression at diagnosis, %  median (range) | 13 (0-98) | 12 (0.5-98) | 21 (0-95) | .47 | 9 (0.5-89) | .06 |
| BM CD61 expression at diagnosis, %  median (range) | 5 (0.5-72) | 5 (0.5-53) | 4 (0.5-72) | .68 | 6 (0.5-67) | .34 |
| BM CD45 expression at diagnosis, %  median (range) | 90 (13-100) | 90 (42-100) | 93 (13-100) | .04 | 87 (29-99) | .008 |
| **HSCT-related parameters** | | | | | | |
| Age at HSCT, years  median (range) | 63.1 (21.4-76.8) | 64.6 (33.1-75.3) | 63.0 (31.3-76.8) | .41 | 61.8 (21.4-75.9) | <.001 |
| Remission status prior to HSCT  CR  CRi  active disease | 211 (54)  51 (13)  130 (33) | 116 (79)  31 (21)  0 (0) | 95 (83)  20 (27)  0 (0) | .53 | 0 (0)  0 (0)  130 (100) | <.001 |
| Donor type, n (%)  related  unrelated, HLA matched  HLA mismatched  haploidentical | 46 (12)  261 (67)  81 (21)  4 (1) | 18 (12)  92 (63)  37 (25)  0 (0) | 13 (11)  79 (62)  23 (20)  0 (0) | .57 | 15 (12)  90 (69)  21 (16)  4 (3) | .02 |
| Conditioning regimen, n (%)  RIC  NMA | 102 (26)  290 (74) | 6 (4)  141 (96) | 7 (6)  108 (94) | .57 | 89 (68)  41 (32) | <.001 |
| HCT-CI Score, n (%)  0  1 or 2  ≥ 3 | 153 (60)  63 (25)  38 (15) | 53 (55)  24 (25)  19 (29) | 48 (61)  21 (27)  10 (13) | .46 | 52 (66)  18 (23)  9 (11) | .43 |
| CMV status, n (%)  recipient + / donor –  all others | 153 (39)  235 (61) | 66 (45)  80 (55) | 38 (33)  77 (67) | .05 | 49 (39)  78 (61) | .81 |
| aGvHD ≥ grade 2, n (%)  absent  present | 234  107 | 87 (70)  37 (30) | 63 (61)  41 (39) | .16 | 84 (74)  29 (26) | .14 |
| cGvHD, n (%)  absent  limited  extended | 96 (39)  36 (15)  113 (46) | 28 (27)  21 (20)  55 (53) | 27 (35)  9 (12)  42 (54) | .23 | 41 (65)  6 10)  16 (25) | <.001 |
| Donor sex*,* n (%)  female into male  all others | 54 (14)  335 (86) | 26 (18)  121 (82) | 11 (10)  102 (90) | .08 | 17 (13)  112 (87) | .88 |
| *Abbreviations: ASXL1, additional sex combs-like 1 gene; BM, bone marrow CEBPA, CCAAT/enhancer-binding protein alpha gene; CMV, cytomegalie virus; CR, complete remission; CRi, complete remission with incomplete peripheral recovery; ELN2017, European Leukemia Net 2017; FLT3-ITD, internal tandem duplication of the FLT3 gene; Hb, hemoglobin; HLA, human leukocyte antigen;HCT-CI, hematopietic cell transplantation comorbidity index; HSCT, hematopoietic stem cell transplantation; NMA, non-myeloablative conditioning; NPM1, nucleophosmin 1 gene; PB, peripheral blood; RIC, reduced intensity conditioning; RUNX1, Runt-related transcription factor 1 gene; TP53, tumor protein 53 gene; WBC, white blood count.* | | | | | | |

**Supplementary Table S2.** Multivariate analyses for the whole patient cohort.

|  | Cumulative incidence of relapse/progression | | Cumulative incidence of non-relapse mortality | | Overall survival | | Event-free survival | |
| --- | --- | --- | --- | --- | --- | --- | --- | --- |
|  | **HR* (95% CI)** | ***P*** | **HR* (95% CI)** | ***P*** | **OR** (95% CI)** | ***P*** | **OR** (95% CI)** | ***P*** |
| ELN2017 genetic risk  (adverse *vs* intermediate *vs* favorable) | 1.54 (1.19-1.99) | .001 | - | - | 0.68 (0.56-0.81) | <.001 | 0.59 (0.46-0.76) | <.001 |
| Pre-HSCT remission status  (active disease *vs* MRD^pos^ *vs* MRD^neg^) | 1.48 (1.20-1.84) | <.001 | - | - | 0.66 (0.54-0.81) | <.001 | 0.58 (0.47-0.73) | <.001 |
| Number of CR/CRi  (none *vs* first *vs* second) | - | - | 0.66 (0.48-0.91) | .01 | - | - | - | - |
| Age at HSCT | - | - | 1.02 (1-1.05) | .05 | - | - | - | - |
| *Abbreviations: AML, acute myeloid leukemia; CI, confidence interval; CR, complete remission; CRI, CR with incomplete peripheral recovery; ELN2017, European LeukemiaNet 2017; HSCT, hematopoietic stem cell transplantation; MRD, measurable residual disease.*  *HR, hazard ratio, <1 (>1) indicate lower (higher) risk of relapse for the first category listed for the dichotomous variables for the lower (higher) values of the continuous variables.  **OR, odds ratio, <1 (>1) indicate lower (higher) chance of survival for the first category listed for the dichotomous variables.  Variables considered in the models were those significant at α=0.10 in univariable analyses.  For CIR endpoint, variables considered were: ELN2017 genetic risk group, conditioning regimen (NMA *vs* RIC) and pre-HSCT remission status (active disease *vs* MRD^pos^ *vs* MRD^neg^). For NRM endpoint, variables considered were age at HSCT, number of CR/CRi (none cs first vs second), and donor type (matched related *vs* matched unrelated *vs* mismatched unrelated). For OS endpoint, variables considered were ELN2017 genetic risk group, donor type (matched related *vs* matched unrelated *vs* mismatched unrelated), and pre-HSCT remission status (active disease *vs* MRD^pos^ *vs* MRD^neg^). For EFS endpoint, variables considered were ELN2017 genetic risk group, conditioning regimen (nma *vs* ric), number of CR/CRi (none cs first vs second), and pre-HSCT remission status (active disease *vs* MRD^pos^ *vs* MRD^neg^). | | | | | | | | |

**Supplementary Table S3.** Multivariate landmark analyses for patients surviving longer than 100 days after RIC- or NMA-HSCT (n=245).

|  | Cumulative incidence of relapse/progression | | Overall survival | | Event-free survival | |
| --- | --- | --- | --- | --- | --- | --- |
|  | **HR* (95% CI)** | ***P*** | **OR** (95% CI)** | ***P*** | **OR** (95% CI)** | ***P*** |
| ELN2017 genetic risk  (adv *vs* interm *vs* fav) | 1.58 (1.12-2.22) | .01 | 0.71 (0.55-0.93) | .01 | 0.60 (0.44-0.83) | .002 |
| Pre-HSCT remission status  (active disease *vs* MRD^pos^ *vs* MRD^neg^) | 1.72 (1.27-2.32) | <.001 | 0.75 (0.58-0.96) | .03 | 0.60 (0.44-0.81) | <.001 |
| Chronic GvHD  (present *vs* absent) | 0.59 (0.35-0.99) | .05 | - | - | 3.85 (1.16-3.23) | .01 |
| *Abbreviations: CI, confidence interval; ELN, European LeukemiaNet; GvHD, graft-versus-host disease; HSCT, hematopoietic stem cell transplantation; MRD, measurable residual disease.*  *HR, hazard ratio, <1 (>1) indicate lower (higher) risk of relapse for the first category listed for the dichotomous variables for the lower (higher) values of the continuous variables.  **OR, odds ratio, <1 (>1) indicate lower (higher) chance of survival for the first category listed for the dichotomous variables.  Variables considered in the models were those significant at α=0.10 in univariable analyses.  For CIR endpoint, variables considered were: ELN2017 genetic risk group, conditioning regimen (NMA *vs* RIC), pre-HSCT remission status (active disease *vs* MRD^pos^ *vs* MRD^neg^), age at HSCT and chronic GvHD (presence *vs* absence). For OS endpoint, variables considered were ELN2017 genetic risk group, donor type (matched related *vs* matched unrelated *vs* mismatched unrelated), and pre-HSCT remission status (active disease *vs* MRD^pos^ *vs* MRD^neg^). For EFS endpoint, variables considered were ELN2017 genetic risk group, conditioning regimen (NMA *vs* RIC), pre-HSCT remission status (active disease *vs* MRD^pos^ *vs* MRD^neg^), age at HSCT and chronic GvHD (presence *vs* absence). | | | | | | |

**Supplementary Figures**

**Supplementary Figure S1**

**Supplementary Figure S1.** **Outcome according to remission status prior to allogeneic RIC- or NMA-HSCT (MRD^neg^ *vs* MRD^pos^ *vs* active disease, n=392).** **(A)** Cumulative incidence of relapse/progression, **(B)** Non-relapse mortality, and **(C)** Overall survival.

**Supplementary Figure S2**

**Supplementary Figure S2.** Outcome according to remission status prior to allogeneic HSCT (MRD^neg^ *vs* MRD^pos^ *vs* active disease) in AML patients receiving NMA-HSCT (n=290). **(A)** Cumulative incidence of relapse/progression, **(B)** Overall survival, and **(C)** Event-free survival.

**Supplementary Figure S3**

**Supplementary Figure S3.** Outcome according to applied conditioning regimen (RIC *vs* NMA) in AML patients transplanted with active disease (n=130). **(A)** Cumulative incidence of relapse/progression, **(B)** Overall survival, and **(C)** Event-free survival.

**Supplementary Figure S4**

**Supplementary Figure S4.** Outcome according to remission status prior to allogeneic HSCT (MRD^neg^ *vs* MRD^pos^ *vs* active disease) within the three ELN2017 risk groups. **(A)** Cumulative incidence of relapse/progression, and **(B)** Overall survival in ELN2017 favorable risk patients (n=75); **(C)** Cumulative incidence of relapse/progression, and **(D)** Overall survival in ELN2017 intermediate risk patients (n=88) and **(E)** Cumulative incidence of relapse/progression, and **(F)** Overall survival in ELN2017 adverse risk patients (n=131).

**Supplementary Figure S5**

**Supplementary Figure S5.** Outcome according to the presence of a chronic GvHD in patients surviving longer than 100 days after RIC- or NMA-HSCT (landmark analysis). **(A)** Cumulative incidence of relapse/progression, and **(B)** Overall survival in all patients (n=245); **(C)** Cumulative incidence of relapse/progression and **(D)** Overall survival in MRD^neg^ patients (n=104), **(E)** Cumulative incidence of relapse/progression and **(F)** Overall survival in MRD^pos^ patients (n=78), and **(G)** Cumulative incidence of relapse/progression and **(H)** Overall survival in patients transplanted with active disease (n=63).

**Supplementary Figure S6**

**Supplementary Figure S6.** Outcome according to application or no application of a preemptive DLI in patients receiving FLAMSA-based RIC-HSCT and surviving relapse-free for at least 120 days after HSCT (n=49). **(A)** Cumulative incidence of relapse, **(B)** Overall survival, and **(C)** Event-free survival.

**Supplementary Figure S7**

**Supplementary Figure S6.** Outcome according to remission status prior to allogeneic HSCT (MRD^neg^ *vs* MRD^pos^ *vs* active disease) in patients receiving *in vivo* T-cell depletion (n=94). **(A)** Cumulative incidence of relapse/progression, **(B)** Overall survival, and **(C)** Event-free survival.
